# Polysomnographic and Electromyographic Evaluation of Sleep Bruxism in Young Colombian Adults: Case-Control Study

**DOI:** 10.3390/jcm14186521

**Published:** 2025-09-17

**Authors:** Olga Patricia López-Soto, Juan Alberto Aristizábal-Hoyos, Héctor Fuentes-Barría, Raúl Aguilera-Eguía, Karen Sofia Gallón-Bedoya, Alejandra Ceballos-Montoya, Lissé Angarita-Dávila, Ángel Roco-Videla, Marcela Caviedes-Olmos

**Affiliations:** 1 Departamento de Salud Oral, Facultad de Salud, Universidad Autónoma de Manizales, Caldas 170008, Colombia; sonrie@autonoma.edu.co (O.P.L.-S.); jaristi@autonoma.edu.co (J.A.A.-H.); karens.gallonb@autonoma.edu.co (K.S.G.-B.); alejandra.ceballosm@autonoma.edu.co (A.C.-M.); 2 Vicerrectoría de Investigación e Innovación, Universidad Arturo Prat, Iquique 1110939, Chile; hefuentes_@unap.cl; 3 Escuela de Odontología, Facultad de Odontología, Universidad Andres Bello, Concepción 3349001, Chile; 4 Departamento de Salud Pública, Facultad de Medicina, Universidad Católica de la Santísima Concepción, Concepción 3349001, Chile; raguilerae@ucsc.cl; 5 Escuela de Nutrición y Dietética, Facultad de Medicina, Universidad Andres Bello, Concepción 3349001, Chile; lisse.angarita@unab.cl; 6 Dirección de Desarrollo y Postgrados, Universidad Autónoma de Chile, Galvarino Gallardo 1983, Santiago 7500138, Chile; angel.roco@uautonoma.cl; 7 Facultad de Salud y Ciencias Sociales, Universidad de las Américas, Providencia, Santiago 7500975, Chile

**Keywords:** sleep bruxism, temporomandibular joint, physical examination, adult, case–control studies

## Abstract

**Background**: Sleep bruxism (SB) is increasingly recognized not merely as a movement disorder but as a multifactorial condition in which physiological, behavioral, and contextual factors converge. **Objective**: To comprehensively characterize SB in young adults, integrating polysomnography (PSG) and surface electromyography (sEMG) to describe sleep architecture, periodic limb movements (PLMs), and masticatory muscle activity; compare these parameters with matched controls; and explore clinical correlations relevant to dental practice and individualized management. **Methods**: Forty university adults (20 PSG-confirmed SB; 20 controls) underwent PSG assessment of total sleep time, sleep stages, arousals, apnea, oximetry, and PLMs. EMG activity of the masseter and temporalis muscles was recorded in 37 participants (18 SB, 19 controls). Statistical analyses included *t*-tests, Mann–Whitney U tests, and multivariate logistic regression to identify independent predictors of SB. **Results**: SB participants exhibited higher bruxism event counts (*p* ≤ 0.001; PS = 0.94), increased PLMs (*p* ≤ 0.01; PS = 0.75), shorter REM sleep duration (*p* = 0.04; d = 0.69), and higher bruxism-related arousal indices (*p* ≤ 0.001; PS = 83.4). Left masseter activity differed significantly (*p* = 0.03; d = 0.50), while other muscle measures showed no significant differences. Logistic regression identified age (OR = 0.59, *p* = 0.02), PLMs (OR = 0.96, *p* = 0.03), and REM sleep duration (OR = 0.98, *p* = 0.05) as independent predictors, explaining 58% of the variance. **Conclusions**: These findings provide a comprehensive profile of SB in young adults. Integrating PSG, sEMG, and oral assessments supports early diagnosis, personalized management, and interdisciplinary collaboration to prevent complications.

## 1. Introduction

Sleep bruxism (SB) has traditionally been classified as a sleep-related movement disorder characterized by repetitive, involuntary contractions of the masticatory muscles during nighttime rest. These manifestations are mainly expressed as rhythmic masticatory muscle activity or as sustained tonic activity [1,2]. However, in recent years, it has been proposed that SB should not be understood solely as a movement disorder, but rather as a condition with its own characteristics, in which physiological, behavioral, and contextual factors converge [3].

In this context, SB is clinically significant due to its high prevalence in the general population—estimated between 8% and 31% in children and young adults and its association with a spectrum of adverse orofacial outcomes, including abnormal tooth wear, masticatory muscle hypertrophy, temporomandibular disorders, myofascial pain, headaches, and reduced quality of life [4,5,6,7]. Despite extensive research, the precise etiology of SB remains multifactorial and incompletely understood, involving a complex interplay of central nervous system mechanisms, sleep microstructure alterations, autonomic dysfunction, and peripheral muscle activity [8,9].

Clinically, SB diagnosis has traditionally relied on a combination of self-reported symptoms, such as teeth grinding or clenching during sleep, bed partner observations, and clinical signs including dental wear facets and masticatory muscle hypertrophy [10]. However, these approaches are limited in sensitivity and specificity, potentially leading to misclassification or overdiagnosis. Consequently, polysomnography (PSG) combined with electromyographic (EMG) recordings of the masseter and temporalis muscles has emerged as the gold standard for objective SB diagnosis and quantification [5,6]. PSG enables simultaneous assessment of muscle activity, sleep stages, micro-arousals, and respiratory events, providing an integrated understanding of the neurophysiological underpinnings of the disorder.

Physiopathologically, SB is hypothesized to be closely linked to sleep fragmentation and arousals, during which brief cortical or autonomic activations trigger rhythmic masticatory muscle activity episodes [8,11,12]. These micro-arousals, particularly during Non- Rapid Eye Movement (N-REM) sleep, have been implicated in initiating SB events. Additionally, alterations in Rapid Eye Movement (REM) sleep duration and architecture may modulate SB occurrence and severity, although studies report heterogeneous findings [13,14,15]. Periodic limb movements during sleep (PLMs), commonly observed in restless legs syndrome and other sleep disorders, have also been found to cooccur with SB, suggesting shared neural circuits involved in sleep-related motor control [15,16]. The relationship between PLMs and SB remains incompletely elucidated, with some evidence indicating that increased PLMs may exacerbate sleep fragmentation and SB activity.

Electromyographic assessment during wakefulness and sleep provides crucial insights into neuromuscular function in SB patients. Surface EMG (sEMG) of the masseter and temporalis muscles allows quantification of activation levels and asymmetries, potentially reflecting underlying hyperactivity, neuromuscular imbalances, or compensatory mechanisms in response to chronic overuse [17,18,19]. However, research exploring these muscle characteristics in young populations with PSG-confirmed SB is limited, representing a significant gap in understanding the peripheral manifestations of this central sleep disorder.

Given the multifactorial nature of SB, integrative studies combining objective polysomnographic data, oral functional assessments, and electromyographic profiles are needed to unravel the complex interactions contributing to SB pathogenesis and clinical expression [5,8,16]. Identifying independent predictors of SB among neurophysiological and demographic variables could enhance diagnostic accuracy and inform personalized therapeutic approaches [8,13]. Moreover, clarifying the roles of age, REM sleep duration, and PLMs in SB physiopathology may provide insights into modifiable factors and risk stratification [13,14,15].

From a clinical perspective, findings derived from PSG and electromyography EMG provide a valuable opportunity to enhance routine dental practice. Whereas traditional assessments rely on indirect signs such as tooth wear facets or muscle hypertrophy, the incorporation of these objective methods allows clinicians to identify specific patterns of masticatory activity, quantify their intensity, and relate them to sleep architecture. For dentists, this means the ability to distinguish clinically significant sleep bruxism from benign manifestations, thereby avoiding overdiagnosis and unnecessary treatments. Moreover, integrating PSG/EMG data into routine evaluations may support more personalized decisions, such as tailoring occlusal splints based on the patient’s electromyographic profile, identifying risks of progression toward temporomandibular disorders, and making timely referrals to sleep medicine specialists when systemic alterations are detected.

For these reasons, this case–control study aimed to comprehensively characterize sleep bruxism in young adults, conceptualized as a sleep-related masticatory muscle activity rather than a disorder. Specifically, we sought to (i) integrate PSG and sEMG data to describe sleep architecture, periodic limb movements, and masticatory muscle activity; (ii) compare these parameters between PSG-confirmed sleep bruxism participants and matched controls; and (iii) explore potential clinical correlations relevant to dental practice and individualized management.

## 2. Materials and Methods

### 2.1. Study Design

This case–control study was conducted in accordance with the STROBE guidelines for observational research [20]. The protocol was initially approved by the Ethics Committee of the Autonomous University of Manizales (Protocol Code: 59-2016; approved on 14 September 2016). Subsequently, the same committee granted retrospective approval (Protocol Code: 292-210; approved on 25 August 2025), which corresponded to an administrative requirement to revalidate the secondary use of data already collected in 2016 for analysis and publication purposes. No new data collection or patient contact occurred after the original approval, and all procedures complied with the Colombian Ministry of Health Resolution 8430 of 1993 and the principles of the Declaration of Helsinki [21].

### 2.2. Context

The study was conducted within the Oral Health Prevention Program at the Autonomous University of Manizales (Colombia), focusing on a population of young adults aged 18–29 years. Sleep bruxism was assessed using overnight PSG, complemented by a functional oral analysis targeting static and dynamic occlusal parameters, as well as sEMG to evaluate masticatory muscle activity.

### 2.3. Participants

A total of 40 university students were enrolled and allocated as follows:

- Cases: 20 participants with PSG-confirmed SB.
- Controls: 20 participants without PSG evidence of SB.

### 2.4. Eligibility Criteria

Inclusion criteria: Students aged 18–29 years, enrolled since 2016 and 2025, with complete medical and dental records and PSG evaluation.

Exclusion criteria: Active dental treatment, more than four conservative prosthetic restorations, use of medications affecting sleep or motor function, neurological disorders, more than two non-functional edentulous areas, use of removable prostheses, severe malocclusion, or diagnosed psychiatric or neurological conditions.

### 2.5. Sleep Bruxism Diagnostic Protocol

The diagnosis followed a three-level protocol developed by the Oral Health Prevention Program, based on sensitivity and specific criteria reported in previous studies [22]:

- Self-reported symptoms: Patient and/or bed partner reports of teeth grinding and morning temporal or masseter muscle pain or fatigue.
- Clinical intraoral assessment: Performed by a certified oral rehabilitator, identifying atypical dental wear facets and masseter hypertrophy during voluntary contraction.
- Polysomnography: Participants positive on the first two levels were referred to the sleep laboratory for overnight PSG, conducted by a physiatrist blinded to the clinical status [23].

### 2.6. Polysomnography

PSG recordings were obtained using the Cadwell Easy III system with 10 mm stainless steel electrodes (Cadwell^®^ 302139-200, Cadwell Industries Inc., Kennewick, WA, USA), following the American Academy of Sleep Medicine guidelines [24,25]. Additionally, polysomnographic recordings (Cadwell Easy III, 2016 version; Cadwell Industries Inc., Kennewick, WA, USA) were included to control for sleep-related variables. The PSG protocol included electrooculography, electromyography, airflow measurement, respiratory effort, snoring, cardiac monitoring, oxygen saturation, and body position tracking. EEG channels were also recorded to identify sleep stages. Electrodes were applied with conductive gel, maintaining impedance <5 kΩ, and signals were digitized at ≥500 Hz with 12–16-bit resolution.

Ocular movements were monitored via EOG with mastoid-referenced electrodes in standard positions; movements with latency <500 ms were considered valid, enabling accurate REM sleep phase identification and transitions. Sleep bruxism was diagnosed if ≥2 RMMA episodes/hour or ≥4 muscle events/hour (phasic, tonic, or mixed) were observed, with a BEI >2 [24,25].

### 2.7. Masticatory Electromyography

sEMG was recorded using a Sierra^®^ Wave^®^ system (Cadwell^®^) at a 76.8 kHz sampling rate, with a 10–10,000 Hz band-pass filter and a 200 µV gain, employing 10 mm stainless steel Cadwell^®^ electrodes (302139-200) with conductive gel (Cadwell^®^ 202153-000) [19,26]. The ground electrode was positioned 2 cm above the nasion. A bipolar configuration was applied to evaluate variations in muscle activation [27].

Electrode placement was conducted as follows:

- Masseter muscles: Subjects were asked to close their teeth with maximal force. The active electrode was positioned on the motor point, and the reference electrode was placed 1 cm below the auricular lobe.
- Temporalis muscles: Electrodes were positioned over the anterior belly after maximal activation.

Although SENIAM guidelines were originally developed for limb muscles, the procedures adopted in this study are consistent with their general principles to ensure reproducibility in craniofacial sEMG [28]. Impedance values complied with international standards; values below 5 kΩ are considered acceptable for reliable sEMG recordings [28,29]. Participants were seated upright with the Frankfort plane parallel to the floor, as recommended for masticatory EMG protocols to minimize postural bias [30]. Participants were seated upright against the back of a chair with their arms relaxed, feet on the floor, eyes open, and looking at a point in front of them. Prior to electrode placement, faces were cleaned with 95% ethanol, and conductive gel was applied to reduce low-conductance artifacts [28,31].

The protocol included voluntary contraction tasks, including lip compression, tongue thrust, masticatory contraction, and swallowing. Each measurement was repeated three times, with 30–60 s intervals to reduce fatigue [28,31]. Maximal voluntary contraction (MVC) of the orbicularis oris muscle was determined by 10 s of pursed lips. Nine swallows of 25 mL of water were requested (three per muscle), and participants were instructed to “swallow as usual” while holding the liquid in the mouth until resting. Total elapsed time during the sEMG process did not exceed 10 min. Force was recorded using a Pounds Myoescanner (Neilco Technology, USA), calibrated to 0.0 lb before each session; measurements ≥0.6 lb were considered normal.

Technical specifications of the recording system are summarized as follows:

- Sampling rate: 76.8 kHz;
- Bandwidth: 10–10,000 Hz;
- Gain: 200 µV;
- Input impedance: >100 MΩ for Cadwell^®^ amplifiers;
- Common mode rejection ratio (CMRR): >100 dB;
- Input range: ±5 mV;
- Baseline noise: <1 µV RMS.

Signal acquisition and initial processing were conducted using the Cadwell^®^ Sierra^®^ Wave^®^ software. Electrode and sensor application and verification were performed by a certified technologist, with all procedures logged in a standardized checklist.

Asymmetry indices were calculated as follows [32]:

Masseter Asymmetry (%):

((Right Masseter − Left Masseter)/(Right Masseter + Left Masseter)) × 100

Temporal Asymmetry (%):

((Right Temporal − Left Temporal)/(Right Temporal + Left Temporal)) × 100

The asymmetrical results of the masseter and temporal muscles can show positive values when activity is greater on the right side, and negative values when activity predominates on the left side. A value of 0 indicates balance between both sides [32].

### 2.8. Bias

Potential selection (complete records only), information (self-reported symptoms), and observer (clinical assessments) biases were minimized through; Probabilistic sampling; Blinded evaluators; Standardized protocols and strict inclusion criteria [33]. Nevertheless, a potential geographic bias must be acknowledged, as the sample was restricted to university students from a single urban region, which may limit the generalizability of the findings to broader or more diverse populations [34].

### 2.9. Sample Size

The sample size was estimated using Epi Info™ v7.2 (StatCalc, CDC, Atlanta, GA, USA), a validated software widely used for epidemiological and clinical research due to its robust statistical algorithms and accessibility [35]. The calculation was based on a target population of university students aged 18–29 years, aligning with the demographic profile most frequently associated with SB in Colombian adults is most frequently described [22]. Following recommendations for case–control studies, parameters were set considering an expected prevalence of exposure of 60% in cases and 10% in controls, assuming a 1:1 case–control ratio, an odds ratio (OR) of 2, a 95% confidence interval (CI), and 80% statistical power [22,36,37,38].

This approach ensures adequate sensitivity to detect clinically meaningful associations while minimizing the risk of type II error, the required sample size was 40 participants, consistent with methodological standards that highlight the importance of balancing feasibility and statistical rigor in observational studies with restricted populations [33,34]. Moreover, the incorporation of a relatively high estimated prevalence in the case group was justified by previous reports documenting elevated rates of SB and parafunctional behaviors in young adults under academic stress, thereby increasing the plausibility of detecting group differences [22]. Thus, the final sample comprised 20 confirmed SB cases and 20 matched controls.

### 2.10. Statistical Analysis

Data were analyzed using SPSS v27 (IBM Corp., USA). Normality was assessed via the Shapiro–Wilk test; normally distributed variables were compared with *t*-tests, and non-normal variables with the Mann–Whitney U test. Means ($\bar{x}$), standard deviations (SD), odds ratios (OR), and 95% confidence intervals (CI) were reported. Effect sizes were estimated using the probability of superiority (PS) for non-parametric data (null ≤ 0.55; small > 0.56; medium > 0.64; large > 0.71; very large > 0.80) and Cohen’s d for parametric data (small 0.1; medium 0.3; large ≥ 0.7) [39,40].

A binary logistic regression model was constructed to identify significant predictors of sleep bruxism. Independent variables included left masseter activity, REM sleep duration, and age, selected based on prior significant differences or trends. Variables directly defining bruxism events were excluded to avoid redundancy and collinearity. Regression coefficients (B), ORs with 95% CIs, and associated *p*-values were reported. Statistical significance was set at *p*-value < 0.05.

## 3. Results

Table 1 provides a descriptive and comparative summary of PSG variables, physiological activity, and specific events in participants with sleep bruxism (*n* = 20) and controls (*n* = 20). Participants SB exhibited several clinically relevant differences compared with matched controls. SB participants were slightly older (mean 22.85 vs. 20.35 years; *p* = 0.01, large effect size), which may reflect developmental or behavioral factors associated with sleep-related masticatory activity. REM sleep duration was significantly longer in the SB group (123.72 vs. 93.97 min; *p* = 0.04, moderate effect size), while Stage 1 sleep was reduced (9.45 vs. 18.10 min; *p* = 0.02, moderate effect size), suggesting alterations in sleep architecture that may facilitate the occurrence of bruxism episodes.

PLMs were notably higher in the SB group (84.20 vs. 49.70 counts; *p* ≤ 0.01, moderate-to-large effect size), highlighting a possible shared central nervous system mechanism influencing both PLMs and SB. Most strikingly, bruxism events were markedly increased in SB participants across total, NREM, and REM sleep, as well as in arousals (*p* ≤ 0.001, very large effect sizes >0.90), confirming the severity of masticatory muscle activity in this population.

Other sleep parameters—including total sleep time, NREM sleep duration, apneas, hypopneas, oxygen saturation, and heart rate—did not differ significantly, indicating that SB in young adults is primarily associated with altered sleep architecture, heightened motor activity, and increased bruxism events, rather than cardiorespiratory disturbances. These findings reinforce the clinical relevance of monitoring SB through PSG and EMG, allowing early identification of patients at risk for dental wear, temporomandibular disorders, and other sequelae.

Of the initial 40 participants, 9 (23%) were men and 31 (77%) were women. Following attrition during follow-up (2 in the bruxism group and 1 in the control group), the bruxism group comprised 5 men (38.5%) and 13 women (61.5%), with a mean age of 22.72 ± 3.87 years. The control group included 4 men (21.05%) and 15 women (79.95%), with a mean age of 20.42 ± 1.57 years.

Table 2 provides EMG analysis of the masseter and temporalis muscles showed moderate differences between participants with SB and controls. While the average activity of the masseter and temporalis muscles did not differ significantly in most measures, the left masseter showed higher activity in the SB group (*p* = 0.03, moderate effect size = 0.50), suggesting a pattern of unilateral overuse in some patients.

Additionally, masseter muscle asymmetry tended to be greater in SB participants (very large effect size = 1.07), although it did not reach statistical significance. Clinically, this may reflect chronic neuromuscular imbalances associated with increased masticatory activity during sleep. Temporal muscle activity and asymmetry did not show relevant differences, indicating that peripheral changes in SB are primarily localized to the masseter, likely due to its central role in generating force during bruxism episodes.

These findings highlight the clinical importance of assessing muscle activity and symmetry in SB patients, as masseter alterations may predispose to hypertrophy, myofascial pain, or temporomandibular disorders, and guide individualized management strategies such as customized occlusal splints targeting patterns of muscular overuse.

Table 3 presents the mean resting electromyographic activity of the masseter muscle.

Analysis of the mean resting contractile force of the masseter muscles showed no statistically significant differences between participants with SB and controls (*p* > 0.05 for all comparisons), with effect sizes around 0.50 or lower. Both right and left masseters exhibited similar resting activity, and asymmetrical values were minimal in both groups.

Clinically, these findings suggest that resting masseter tone is not altered in young adults with SB, indicating that the peripheral muscular changes observed during sleep (e.g., increased EMG activity and asymmetry during bruxism events) do not persist at rest. This distinction is important for clinical evaluation, as it implies that interventions should focus on sleep-time muscle hyperactivity rather than baseline muscle tone, guiding decisions for occlusal splints, physiotherapy, or targeted biofeedback strategies.

To identify predictors of sleep bruxism, a multivariate logistic regression analysis was performed (Table 4). Multivariate logistic regression analysis identified age, PLMs, and REM sleep duration as factors associated with SB in young adults. Higher age was a significant predictor of lower odds of SB (B = −0.53; OR = 0.59; 95% CI: 0.39–0.90; *p* = 0.02), suggesting that younger adults are more prone to develop clinically relevant bruxism.

Interestingly, increased PLMs was slightly associated with reduced likelihood of SB (B = −0.04; OR = 0.96; 95% CI: 0.92–1.00; *p* = 0.03), indicating a subtle interplay between central motor activity and masticatory events during sleep. Longer REM sleep duration showed a similar trend, although marginally non-significant (B = −0.02; OR = 0.98; *p* = 0.05), implying that REM architecture may modulate bruxism expression, potentially through its effect on arousal thresholds and muscle activation.

The regression model demonstrated good fit (global χ^2^ = 32.51, *p* < 0.001; Nagelkerke R^2^ = 0.58), explaining approximately 58% of the variability in SB occurrence. Clinically, these findings highlight that age and sleep-related motor patterns should be considered when assessing bruxism risk, supporting the use of PSG and EMG for individualized evaluation and early identification of patients at higher risk for dental and temporomandibular complications.

## 4. Discussion

This case–control study, based on PSG and sEMG, provides robust evidence of the physiological and neuromuscular patterns that distinguish individuals with SB from healthy controls in a cohort of young adults. Our findings confirm that SB is associated with significant alterations in sleep architecture, a higher prevalence of PLMs, and a substantial increase in nocturnal masticatory activity, both in REM and N-REM phases. Independent predictors of SB were also identified, highlighting the role of age, REM sleep duration, and peripheral motor activity during sleep.

PSG data revealed a significantly higher frequency of SB events in the case group, with indices far exceeding the clinical threshold of ≥2 episodes of rhythmic masticatory muscle activity per hour as proposed by the American Academy of Sleep Medicine [25,26]. This nocturnal muscular hyperactivity, often accompanied by arousals, supports the hypothesis that SB represents a dysrhythmic sleep pattern in which brief autonomic and cortical events play a central role as triggers [8,11,12]. Consistently, our results showed an increase in stage 1 sleep—usually considered light and less restorative—and a relative reduction in total REM sleep, in line with previous studies linking SB to a fragmented and less restorative sleep architecture [13,14,15].

A particularly relevant finding was the higher frequency of PLMs in the SB group, suggesting a potential shared dysfunction in neuromotor mechanisms underlying motor control during sleep. While previous studies have proposed a coexistence of PLMs and SB, our analysis identified PLMS as a negative predictor of SB (OR = 0.96; *p* = 0.03), indicating that higher PLMs was slightly associated with lower odds of bruxism. This inverse relationship is intriguing and may reflect a neurophysiological competition between different motor circuits during sleep, where activation of leg motor pathways could transiently inhibit masticatory muscle bursts. Alternatively, PLMs might act as modulators of arousal thresholds, influencing the timing or intensity of bruxism episodes [16]. Further research using longitudinal designs, neuroimaging, and connectivity studies is warranted to clarify these mechanisms, which could provide valuable insights for individualized management and interdisciplinary collaboration between dentists, neurologists, and sleep specialists.

Regarding age, our results showed that SB was more prevalent among younger participants, consistent with prior literature indicating a progressive decline in bruxism activity with aging [4,5,6]. The multivariate logistic regression confirmed this pattern, revealing an inverse association between age and the likelihood of SB (OR = 0.59; *p* = 0.02), underscoring the need for early detection strategies in younger populations, particularly in university settings where psychosocial stress levels are high.

Electromyographic analysis revealed significant differences in left masseter activity during functional contraction, with a trend toward neuromuscular asymmetry, although this did not reach statistical significance. The observed unilateral predominance could reflect chronic functional adaptation or preferential lateralization in chewing and bruxism patterns, as previously reported in functional imaging and EMG studies [17,18,19,32]. However, masseter resting activity did not differ significantly between groups, suggesting that muscular hyperactivity in SB is predominantly nocturnal and does not persist during wakefulness.

From a methodological standpoint, the combined use of PSG and sEMG represents a major strength [19,41], enabling an objective and multidimensional characterization of SB. The inclusion of detailed polysomnographic variables, such as respiratory events, arousals, and oximetry, allowed us to control for potential confounding by sleep-disordered breathing, which often complicates SB diagnosis in less rigorous studies. Furthermore, the estimation of effect sizes using probability of superiority and Cohen’s d provided a quantitative appraisal of the clinical impact of the observed differences, beyond statistical significance [39,40].

## 5. Limitations and Clinical Implications

This study has several limitations that should be acknowledged. Although the sample size was statistically calculated, participants were restricted to university students, which may limit the generalizability of the findings to other populations. Moreover, while the case–control design is valuable for exploring significant associations, it does not allow for causal inferences [33]. Future longitudinal research could expand on these results by examining the progression of SB in relation to changes in sleep architecture, psychosocial factors, and neuroplasticity. In addition, integrating functional neuroimaging and cortical connectivity analyses may provide further insights into the neural circuits underlying the pathophysiology of SB.

A further limitation concerns the non-evaluation of psychological factors. Recent evidence indicates that the type of sleep bruxism activities can be influenced by psychological distress, and the omission of this assessment—particularly in a small sample—could have introduced an important bias [42]. Indeed, the Standardized Tool for the Assessment of Bruxism (STAB) recommends evaluating psychological parameters in conjunction with bruxism activity. Another limitation relates to the accuracy of sleep timing. Even a mismatch of 20 min determining when participants fell asleep can influence the total masticatory muscle activity [43]. This emphasizes the need for precise sleep onset monitoring in future studies. Finally, given the small sample size and the natural variation in electromyographic waveforms during sleep, the findings may have limited external validity. Larger cohorts are required in future research to capture the full range of SB-related activity [44].

The findings of this study have important clinical implications for the diagnosis, monitoring, and management of SB. First, our results reinforce the view of SB as a multifactorial condition with neurophysiological, respiratory, and musculoskeletal components [5,8,16,45]. The identification of age, PLMs, and REM sleep duration as independent predictors suggests that SB cannot be fully understood as a peripheral muscular phenomenon or a simple stress-related response, but rather as a complex disorder involving dysregulation of brain networks responsible for sleep architecture, motor control, and autonomic modulation [46].

The association between reduced REM sleep duration and SB further highlights the need to incorporate sleep architecture parameters into clinical assessment [13]. Given the essential role of REM sleep in emotional consolidation and neurovegetative homeostasis, its alteration in SB patients may contribute not only to motor events but also to psychological comorbidities such as anxiety and mood disorders [47,48]. This underscores the importance of providing more concrete clinical recommendations for dental and sleep practice, such as early referral for PSG/EMG assessment in patients with dental wear, morning masticatory discomfort, or muscle hypertrophy, and integrating findings into individualized patient management plans.

Furthermore, an interdisciplinary approach involving dentists, neurologists, clinical psychologists, and sleep medicine specialists is recommended to provide a comprehensive biopsychosocial evaluation, optimize treatment strategies, and facilitate early detection and monitoring of SB.

Moreover, our results emphasize the relevance of functional assessment of the stomatognathic system as part of routine dental examination, particularly in patients reporting morning orofacial pain, dental wear, or muscle fatigue [49]. Early detection of signs such as joint noises or masticatory asymmetry may serve as indirect indicators of SB, enabling timely interventions ranging from patient education to occlusal, physiotherapeutic, or behavioral therapies [50,51].

In this context, the present study strengthens the evidence supporting the development of individualized assessment and management protocols for SB, based on the integration of clinical, functional, and polysomnographic markers. Such strategies not only optimize therapeutic outcomes but also improve patients’ quality of life by preventing associated complications, including temporomandibular disorders, sleep impairment, and psychosocial consequences.

## 6. Conclusions

The findings of this case-control study demonstrate that sleep bruxism is significantly associated with functional alterations of the stomatognathic system, particularly the presence of joint noises and increased masseter muscle activity, as well as with modified sleep patterns, including reduced REM sleep duration and a higher frequency of periodic limb movements. These variables showed significant predictive capacity in the logistic regression model, suggesting a distinctive clinical and neurophysiological profile in individuals with sleep bruxism.

From a clinical perspective, these findings underscore the importance of a comprehensive evaluation that integrates polysomnographic parameters, electromyographic analysis, and functional clinical examination for the diagnosis and monitoring of sleep bruxism. They also highlight the relevance of an interdisciplinary approach, where dentists, neurologists, and sleep medicine specialists collaborate to design preventive and therapeutic strategies tailored to the patient’s individual pathophysiology. Future longitudinal studies with more representative samples and sex-stratified analyses are needed to clarify the causal role of these factors and to establish more precise and sensitive diagnostic criteria for this highly prevalent condition with substantial functional impact.

## Figures and Tables

**Table 1:** Baseline characteristics of PSG variables in subjects with SB (*n* = 20) and controls (*n* = 20).

Variables	Groups	$\bar{x}$	SD	CI 95%	*p*-Value	Effect Size
Age (years)	SB	22.85	3.68	21.13 to 24.57	0.01 † *	0.88
	Control	20.35	1.57	19.62 to 21.08		
Total Sleep time (min)	SB	389.65	32.48	374.45 to 404.85	0.53 ^U^	0.60
	Control	371.77	60.05	343.66 to 399.88		
REM Sleep time (min)	SB	123.72	45.76	102.31 to 145.14	0.04 † *	0.69
	Control	93.97	39.79	75.35 to 112.60		
N-REM Sleep time (min)	SB	265.95	49.53	242.77 to 289.13	0.53 †	0.17
	Control	278.15	85.85	247.33 to 308.97		
Stage 1 sleep (min)	SB	9.45	11.51	4.06 to 14.84	0.02 ^U^ *	0.67
	Control	18.10	15.31	11.9 to 25.26		
Stage 2 sleep (min)	SB	200.27	52.53	175.69 to 224.86	0.22 ^U^	0.61
	Control	175.08	68.46	143.03 to 207.12		
Stage 3 sleep (min)	SB	56.23	25.80	44.15 to 68.30	0.42 †	0.28
	Control	63.50	25.64	51.49 to 75.50		
Total Arousals (count)	SB	30	23.22	18.63 to 40.37	0.37 ^U^	0.63
	Control	20.60	14.68	13.73 to 27.47		
REM arousals index (arousals/hr)	SB	13.30	6.99	10.03 to 16.57	0.55 ^U^	0.57
	Control	11.40	7.11	8.07 to 14.73		
N-REM arousals index (arousals/hr)	SB	11.15	7.87	7.47 to 14.83	0.37 ^U^	0.62
	Control	8.3	4.51	6.19 to 10.41		
Central Apnea Episodes (count)	SB	0.65	1.31	0.04 to 1.26	0.38 ^U^	0.62
	Control	0.20	0.52	−0.04 to 0.44		
Obstructive Apnea Episodes (count)	SB	1.40	3.79	−0.37 to 3.17	0.78 ^U^	0.59
	Control	0.40	0.88	−0.01 to 0.81		
Mixed Apnea Episodes (count)	SB	0.75	1.48	0.06 to 1.44	0.34 ^U^	0.64
	Control	0.20	0.41	0.01 to 0.39		
Hypopnea Episodes (count)	SB	28.95	33.18	13.42 to 44.48	0.25 ^U^	0.59
	Control	19.40	25.72	7.36 to 31.44		
N-REM Oximetry (%)	SB	93.50	1.40	92.85 to 94.15	0.60 ^U^	0.57
	Control	93.15	1.53	92.43 to 93.87		
REM Oximetry (%)	SB	93.85	1.31	93.24 to 94.46	0.84 ^U^	0.59
	Control	89.60	19	80.71 to 98.49		
N-REM heart rate (beats/min)	SB	65.40	6.64	62.29 to 68.51	0.86 †	0.04
	Control	65	11.66	59.54 to 70.46		
REM heart rate (beats/min)	SB	66.80	7.13	63.46 to 70.14	0.83 †	0.06
	Control	66.25	12.30	60.49 to72.01		
Overall heart rate during sleep (mean)	SB	66.05	6.94	62.80 to 69.30	0.73 †	0.09
	Control	65.25	11.31	59.96 to 70.54		
Total PLMs (count)	SB	84.20	45.82	62.76 to 105.64	≤0.01 ^U^ *	0.75
	Control	49.70	20.35	40.18 to 59.22		
PLMs in arousals (count)	SB	6.55	6.72	3.41 to 9.69	0.34 ^U^	0.63
	Control	4.05	3.30	2.51 to 5.59		
Total Bruxism Events (count)	SB	260.50	119	204.80 to 316.20	≤0.001 ^U^ **	0.94
	Control	61.95	39.71	41.37 to 82.51		
N-REM Bruxism Events (count)	SB	135.45	77.81	99.03 to 171.87	≤0.001 ^U^ **	88.1
	Control	37.25	29.52	23.43 to 51.07		
REM Bruxism Events (count)	SB	120.20	90.21	77.98 to 162.42	≤0.001 ^U^ **	82.4
	Control	31.15	32.46	15.96 to 46.34		
Bruxism REM Index (events/hr)	SB	40.60	18.89	31.76 to 49.44	≤0.001 ^U^ **	93.7
	Control	10.14	6.08	7.29 to 12.98		
N-REM Bruxism Index (events/hr)	SB	30.58	15.88	23.15 to 38.01	≤0.001 ^U^ **	91.3
	Control	7.89	5.11	5.49 to 10.28		
Bruxism Events in arousals (count)	SB	86.75	57.03	60.06 to 113.44	≤0.001 ^U^ **	83.4
	Control	28.10	20.35	18.58 to 37.62		

†: *t*-test and Cohen’s d effect size, U: Man’s U test and effect size probability of superiority, $\bar{x}$: mean, SD: Standard deviation, CI: 95% confidence interval. *: significance *p*-value < 0.05, **: significance *p*-value < 0.001.

**Table 2:** Comparison of Masticatory Muscle Activity and Asymmetry of the Masseter and Temporalis Muscles Between SB (*n* = 18) and Control (*n* = 19).

Variable	SB		Control		Inferential Statistics	
	$\bar{x}$ (CI 95%)	SD	$\bar{x}$ (CI 95%)	SD	*p*-Value	Effect Size
Right masseter muscle (lb)	0.04 (0.03 to 0.05)	0.03	0.06 (0.03 to 0.09)	0.05	0.28 U	0.34
Left masseter muscle (lb)	0.08 (−0.03 to 0.19)	0.23	0.07 (0.02 to 0.13)	0.10	0.03 U *	0.50
Asymmetry masseter muscle	0.31 (−0.19 to 0.26)	0.45	−0.16 (−0.37 to 0.05)	0.43	0.20 †	1.07
Right temporal muscle (lb)	0.06 (−0.01 to 0.13)	0.14	0.07 (0.3 to 0.11)	0.09	0.19 U	0.47
Left temporal muscle (lb)	0.08 (−0.03 to 0.19)	2.27	0.05 (0.03 to 0.08)	0.05	0.09 U	0.51
Asymmetry temporal muscle	0.13 (−0.03 to 0.30)	0.33	0.04 (−0.11 to 0.21)	0.32	0.41 †	0.25

†: *t*-test and Cohen’s d effect size, U: Man’s U test and effect size probability of superiority, $\bar{x}$: mean, SD: Standard deviation, CI: 95% confidence interval. *: significance *p*-value < 0.05.

**Table 3:** Mean Contractile Force of the Masseter Muscles in SB (*n* = 18) and Control (*n* = 19).

Variable	SB		Control		Inferential Statistics	
	$\bar{x}$ (CI 95%)	SD	$\bar{x}$ (CI 95%)	SD	*p*-Value	Effect Size
Right masseter muscle (lb)	0.48 (0.43 to 0.54)	0.12	0.49 (0.45 to 0.54)	0.10	0.89 ^U^	0.50
Left masseter muscle (lb)	0.48 (0.42 to 0.53)	0.11	0.47 (0.42 to 0.53)	0.11	0.89 ^U^	0.53
Asymmetry masseter muscle	−0.01 (−0.21 to 0.20)	0.41	−0.04 (−0.24 to 0.15)	0.39	0.78 ^U^	0.08

U: Man’s U test and effect size probability of superiority, $\bar{x}$: mean, SD: Standard deviation, CI: 95% confidence interval.

**Table 4:** Logistic Regression Model Results for Predicting Sleep Bruxism in Young Adults.

Model	B	OR	CI 95%	*p*-Value	R^2^	X^2^	Sig Global
Total PLMs	−0.04	0.96	0.92 to 1.00	0.03	0.58	32.51	<0.001
REM Sleep time	−0.02	0.98	0.95 to 1.00	0.05			
Years	−0.53	0.59	0.39 to 0.90	0.02			
Constant	16.45	13,866,151		0.03			

B: Logistic regression coefficient (Beta), OR: Odds Ratio, CI: Confidence Interval (95%), *p*-value: Statistical significance level, R^2^: Nagelkerke’s R-squared, X^2^: Chi-square statistic of the model, Global Sig: Overall model significance (Chi-square test *p*-value).

## Data Availability

The original contributions presented in this study are included in the article/Supplementary Material. The data from this article will be made available by the authors on reasonable request.

## Supplementary Materials

The following supporting information can be downloaded at: https://www.mdpi.com/article/10.3390/jcm14186521/s1.

## Abbreviations

The following abbreviations are used in this manuscript:

SB	Sleep bruxism
PSG	Polysomnography
EMG	Electromyographic
sEMG	Surface electromyographic
PLMs	Periodic limb movements during sleep
REM	Rapid eye movement
N-REM	Non-rapid eye movement

## References

[1] Manfredini D., Ahlberg J., Lobbezoo F. (2022). Bruxism definition: Past, present, and future-What should a prosthodontist know?. J. Prosthet. Dent..

[2] Xu M., Zhong Z., Zou X., Ouyang Q., Zhang L., Yu B., Yao D. (2020). Eye movements in relation to rhythmic masticatory muscle activities in patients with sleep bruxism. Sleep Med..

[3] Verhoeff M.C., Lobbezoo F., Ahlberg J., Bender S., Bracci A., Colonna A., Dal Fabbro C., Durham J., Glaros A.G., Häggman-Henrikson B. (2025). Updating the Bruxism Definitions: Report of an International Consensus Meeting. J. Oral Rehabil..

[4] Chen X., Ke Z.L., Chen Y., Lin X. (2021). The prevalence of sleep problems among children in mainland China: A meta-analysis and systemic-analysis. Sleep Med..

[5] Bulanda S., Ilczuk-Rypuła D., Nitecka-Buchta A., Nowak Z., Baron S., Postek-Stefańska L. (2021). Sleep Bruxism in Children: Etiology, Diagnosis, and Treatment-A Literature Review. Int. J. Environ. Res. Public Health.

[6] Zieliński G., Pająk A., Wójcicki M. (2024). Global Prevalence of Sleep Bruxism and Awake Bruxism in Pediatric and Adult Populations: A Systematic Review and Meta-Analysis. J. Clin. Med..

[7] Mortazavi N., Tabatabaei A.H., Mohammadi M., Rajabi A. (2023). Is bruxism associated with temporomandibular joint disorders? A systematic review and meta-analysis. Evid. Based Dent..

[8] Thomas D.C., Manfredini D., Patel J., George A., Chanamolu B., Pitchumani P.K., Sangalli L. (2024). Sleep bruxism: The past, the present, and the future-evolution of a concept. J. Am. Dent. Assoc..

[9] Casazza E., Giraudeau A., Payet A., Orthlieb J.D., Camoin A. (2022). Management of idiopathic sleep bruxism in children and adolescents: A systematic review of the literature. Arch. Pediatr..

[10] Beddis H., Pemberton M., Davies S. (2018). Sleep bruxism: An overview for clinicians. Br. Dent. J..

[11] Song J.Y. (2021). Implant complications in bruxism patients. J. Korean Assoc. Oral Maxillofac. Surg..

[12] Matusz K., Maciejewska-Szaniec Z., Gredes T., Pobudek-Radzikowska M., Glapiński M., Górna N., Przystańska A. (2022). Common therapeutic approaches in sleep and awake bruxism-an overview. Neurol. Neurochir. Pol..

[13] Kato T., Higashiyama M., Katagiri A., Toyoda H., Yamada M., Minota N., Katsura-Fuchihata S., Zhu Y. (2023). Understanding the pathophysiology of sleep bruxism based on human and animal studies: A narrative review. J. Oral Biosci..

[14] Fulek M., Wieckiewicz M., Szymanska-Chabowska A., Gac P., Poreba R., Markiewicz-Gorka I., Wojakowska A., Mazur G., Martynowicz H. (2024). Inflammatory Markers and Sleep Architecture in Sleep Bruxism-A Case-Control Study. J. Clin. Med..

[15] Zhu Y., Toyota R., Shiraishi Y., Katagiri A., Yamada M., Higashiyama M., Toyoda H., Lavigne G., Kato T. (2024). Sleep architecture as a candidate for phenotyping sleep bruxism: A narrative physiological review. J. Oral Rehabil..

[16] Kuang B., Li D., Lobbezoo F., de Vries R., Hilgevoord A., de Vries N., Huynh N., Lavigne G., Aarab G. (2022). Associations between sleep bruxism and other sleep-related disorders in adults: A systematic review. Sleep Med..

[17] Yamaguchi T., Mikami S., Maeda M., Saito T., Nakajima T., Yachida W., Gotouda A. (2023). Portable and wearable electromyographic devices for the assessment of sleep bruxism and awake bruxism: A literature review. Cranio.

[18] Ohlmann B., Rathmann F., Bömicke W., Behnisch R., Rammelsberg P., Schmitter M. (2022). Validity of patient self-reports and clinical signs in the assessment of sleep bruxism based on home-recorded electromyographic/electrocardiographic data. J. Oral Rehabil..

[19] Monteiro U.M., Soares V.B.R.B., Soares C.B.R.B., Pinto T.C.C., Ximenes R.C.C., Araújo Cairrão Rodrigues M. (2021). Electromyographic Patterns and the Identification of Subtypes of Awake Bruxism. Front. Hum. Neurosci..

[20] Cuschieri S. (2019). The STROBE guidelines. Saudi J. Anaesth..

[21] World Medical Association (2025). World Medical Association Declaration of Helsinki: Ethical principles for medical research involving human subjects. JAMA.

[22] Aristizabal-Hoyos J.A., López-Soto O., Fuentes-Barría H., Aguilera-Eguía R., Angarita-Davila L., Rojas-Gómez D. (2025). Sleep Bruxism and Occlusal Function: A Case–Control Study Based on Polysomnography in Young Colombians. J. Clin. Med..

[23] Raja H.Z., Saleem M.N., Mumtaz M., Tahir F., Iqbal M.U., Naeem A. (2024). Diagnosis of Bruxism in Adults: A Systematic Review. J. Coll. Physicians Surg. Pak..

[24] Kanclerska J., Wieckiewicz M., Poreba R., Szymanska-Chabowska A., Gac P., Wojakowska A., Frosztega W., Michalek-Zrabkowska M., Mazur G., Martynowicz H. (2022). Polysomnographic Evaluation of Sleep Bruxism Intensity and Sleep Architecture in Nonapneic Hypertensives: A Prospective, Observational Study. J. Clin. Med..

[25] Howell M., Avidan A.Y., Foldvary-Schaefer N., Malkani R.G., During E.H., Roland J.P., McCarter S.J., Zak R.S., Carandang G., Kazmi U. (2023). Management of REM sleep behavior disorder: An American Academy of Sleep Medicine clinical practice guideline. J. Clin. Sleep Med..

[26] López Soto O.P., López Soto L.M., Osorio Forero A., Restrepo de Mejía F. (2014). Lado de preferencia masticatoria en niños con fisura palatina: Concordancia de tres métodos. Rev. Fac. Odontol. Univ. Antioq..

[27] Ocak I., Soylu A.R., Aksu M. (2022). Changes in Orbicularis Oris Superior and Masseter Muscle Activities After Upper Incisor Protrusion in Class II Division 2 Malocclusion: An Electromyographic Study. Turk. J. Orthod..

[28] Hermens H.J., Freriks B., Disselhorst-Klug C., Rau G. (2000). Development of recommendations for SEMG sensors and sensor placement procedures. J. Electromyogr. Kinesiol..

[29] Merletti R., Parker P. (2004). Electromyography: Physiology, Engineering and Non-Invasive Applications.

[30] Gadotti I., Hicks K., Koscs E., Lynn B., Estrazulas J., Civitella F. (2020). Electromyography of the masticatory muscles during chewing in different head and neck postures-A pilot study. J. Oral Biol. Craniofac Res..

[31] López-Soto L.M., López-Soto O.P., Osorio-Forero A., Restrepo F., Tamayo-Orrego L. (2017). Muscle Activity and Muscle Strength in Atypical Swallowing. Salud Uninorte.

[32] Naeije M., McCarroll R.S., Weijs W.A. (1989). Electromyographic activity of the human masticatory muscles during submaximal clenching in the inter-cuspal position. J. Oral Rehabil..

[33] Palumbo S.A., Robishaw J.D., Krasnoff J., Hennekens C.H. (2021). Different biases in meta-analyses of case-control and cohort studies: An example from genomics and precision medicine. Ann. Epidemiol..

[34] Skopec M., Issa H., Reed J., Harris M. (2020). The role of geographic bias in knowledge diffusion: A systematic review and narrative synthesis. Res. Integr. Peer Rev..

[35] Camp B., Mandivarapu J.K., Ramamurthy N., Wingo J., Bourgeois A.G., Cao X., Sunder-raman R. (2018). A new cross-platform architecture for epi-info software suite. BMC Bioin Form..

[36] Serdar C.C., Cihan M., Yücel D., Serdar M.A. (2021). Sample size, power and effect size revisited: Simplified and practical approaches in pre-clinical, clinical and laboratory studies. Biochem. Med..

[37] Shieh G. (2019). Effect size, statistical power, and sample size for assessing interactions between categorical and continuous variables. Br. J. Math. Stat. Psychol..

[38] Cadar M., Almăşan O. (2024). Dental occlusion characteristics in subjects with bruxism. Med. Pharm. Rep..

[39] Uanhoro J.O. (2023). Probability of superiority for comparing two groups of clusters. Behav. Res. Methods..

[40] Zieliński G., Gawda P. (2025). Defining Effect Size Standards in Temporomandibular Joint and Masticatory Muscle Research. Med. Sci. Monit..

[41] Vlăduțu D.E., Ionescu M., Mercuț R., Noveri L., Lăzărescu G., Popescu S.M., Scrieciu M., Manolea H.O., Iacov Crăițoiu M.M., Ionescu A.G. (2022). Ecological Momentary Assessment of Masseter Muscle Activity in Patients with Bruxism. Int. J. Environ. Res. Public Health.

[42] Deregibus A., Graziano V., Bargellini A., Cugliari G., Ravera S., Sanna F., Parrinia S., Castroflorio C., Manfredinie D. (2025). Psychoemotional determinants of sleep bruxism: A phenotypic characterization using instrumental assessment. CRANIO^®^.

[43] Takahashi M., Yamaguchi T., Mikami S., Saito M., Nakajima T., Maeda M., Sakumaa T., Saito T. (2025). Number of masseteric electromyographic waveforms during analysis periods with/without excluding time zones after going to bed and before getting up in sleep bruxism assessment. CRANIO^®^.

[44] Kudo A., Yamaguchi T., Mikami S., Saito M., Nakajima T., Maeda M., Takahashi S., Gotouda A. (2023). Frequency distribution of the number and amplitude of electromyographic waveforms of the masseter muscle during sleep in patients with a clinical diagnosis of sleep bruxism. CRANIO^®^.

[45] Chung J., Lobbezoo F., van Selms M.K.A., Chattrattrai T., Aarab G., Mitrirattanakul S. (2021). Physical, psychological and socio-demographic predictors related to patients’ self-belief of their temporomandibular disorders’ aetiology. J. Oral Rehabil..

[46] Giovanni A., Giorgia A. (2021). The neurophysiological basis of bruxism. Heliyon.

[47] Feriante J., Singh S. (2025). REM Rebound Effect. StatPearls.

[48] Pronier É., Morici J.F., Girardeau G. (2023). The role of the hippocampus in the consolidation of emotional memories during sleep. Trends Neurosci..

[49] Saratti C.M., Rocca G.T., Vaucher P., Awai L., Papini A., Zuber S., Di Bella E., Dietschi D., Krejci I. (2021). Functional assessment of the stomatognathic system. Part 2: The role of dynamic elements of analysis. Quintessence Int..

[50] Lobbezoo F., Ahlberg J., Raphael K.G., Wetselaar P., Glaros A.G., Kato T., Santiago V., Winocur E., De Laat A., De Leeuw R. (2018). International consensus on the assessment of bruxism: Report of a work in progress. J. Oral Rehabil..

[51] López-Soto O.P., Aristizabal-Hoyos J.A., Mulett-Vásquez J., Fuentes-Barría H., Aguilera-Eguía R., Angarita-Davila L., Rojas-Gómez D., Roco-Videla Á. (2025). Psychometric Validation of the Spanish OSAKA Questionnaire: A Cross-Sectional Study Among Colombian Dental Professionals. Dent. J..

